# Hemophagocytic syndrome in pregnancy: case report, diagnosis, treatment, and prognosis

**DOI:** 10.1002/ccr3.1172

**Published:** 2017-09-12

**Authors:** Aline Rousselin, Zarrin Alavi, Emmanuelle Le Moigne, Sarah Renard, Christophe Tremouilhac, Aurélien Delluc, Philippe Merviel

**Affiliations:** ^1^ Obstetrics and Gynecology Service Brest Medical University Hospital Brest France; ^2^ Inserm CIC 1412 Brest Medical University Hospital Brest France; ^3^ Department of Internal Medicine Brest Medical University Hospital Brest France

**Keywords:** Case report, diagnosis, hemophagocytic syndrome, oocyte donation, pregnancy

## Abstract

Diagnosis of hemophagocytic syndrome remains a challenge in particular during pregnancy. Concomitant presence of clinical and biological signs, for example, fever, pancytopenia, hyperferritinemia, and hypertriglyceridemia, should alert clinicians to suspect HPS and proceed to prompt treatments.

## Introduction

Macrophage activating syndrome is also called hemophagocytic syndrome (HPS). It may be primary as in familial hemophagocytic lymphohistiocytosis (HLH), Chediak–Higashi, Griscelli, and Purtilo syndromes, or secondary as in case of malignancies hemato‐oncology, oncologic diseases, infectious diseases, or autoimmune disease. HPS is rare but underdiagnosed and can be life‐threatening if undiagnosed. Its incidence is estimated between 0.8% and 4% cases per year including pediatric and adult HPS 1.

Hemophagocytic syndrome is known since the 1950s, and further described by Risdall et al. 2. The pathophysiology consists of activation of T lymphocytes and natural killer cells (HLH), either secondary to an opportunistic infection, or primary due to a deficiency of immunomodulatory mechanisms.

This HLH immune activation leads to a high production of pro‐inflammatory cytokines. These cytokines activate the monocyte–macrophage system and enhance the HLH in a positive feedback 3, 4, 5, 6, 7.

These macrophages are responsible for hemophagocytosis expressed clinically by various symptoms such as fever, lymphadenopathy, hepatosplenomegaly. Clinical presentation of these signs strongly suggests HPS.

Hemophagocytic syndrome occurrence in pregnancy is rare and there are only a few reported cases in the literature 5, 8, 9, 10, 11, 12, 13, 14, 15, 16, 17, 18, 19. Here, we describe a case of HPS during the third trimester (30 weeks of gestational age (GA)) of pregnancy: its diagnosis, treatment, and fetal and maternal outcomes. This case is further compared with the literature to set forth a proposal for advancement of best clinical practices in HPS during pregnancy.

## Methods

We present here the case of a primigravida 44‐year‐old woman who presented at 30 weeks GA + 4 days to the emergency room for fever of 39.4°C associated with a cough since 15 days. This patient had a history of primary infertility salpingectomy for hydrosalpinx. Raynaud syndrome with positive antinuclear antibodies (e.g., antiribonucleoproteins) and moderate peripheral thrombocytopenia have been diagnosed since 2 years. Antiphospholipid antibodies were negative. The pregnancy was achieved by in vitro fertilization with oocyte donation. Despite abnormal (i.e., dark circles on the legs and arms) skin pigmentation early in pregnancy, the diagnosis of lupus (i.e., before pregnancy onset, she has been followed up for thrombocytopenia and suspicion of autoimmune disease) or Sharp syndrome (mixed connective tissue disease) could not be confirmed. Nevertheless, given the suspicion of autoimmune disease, aspirin 75 mg/day was started.

**Figure 1:**
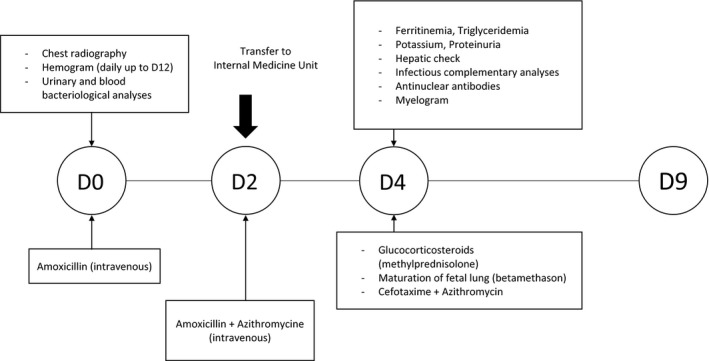
The investigative approaches and initial treatments.

At the emergency room, the patient presented with fever of 39.4°C, blood pressure at 99/62 mmHg, heart rate at 121/min, and oxygen saturation at 98% on room air. There was no history of infection or recent travel. The chest radiography showed some pulmonary infiltrates. Other clinical examinations were normal except for the presence of submandibular adenopathy. Blood biology workup showed moderate pancytopenia and inflammatory syndrome (Table 1). The fetal heart rate recording showed tachycardia (i.e., due to the high fever, 170 beats per min). As a result, the patient was hospitalized in gynecology–obstetrics unit (Fig. 1). Intravenous antibiotic (amoxicillin 1 g tid) was started and the baseline laboratory workups (urinary and blood bacteriological analyses) were negative.

**Table 1:** Laboratory trends from baseline to Day 9

	D0	D2	D3	D4	D9
Temperature (°C)	39.4	38.2	–	36.1	37
Hemoglobin (g/dL)	8.4	8.6	9.3	7.9	9
Platelets (Giga/L)	130	107	103	85	74
Leukocytes (Giga/L)	3.4	2	2.1	–	3.1
Lymphocytes (Giga/L)	–	0.37	0.58	–	0.73
Polynuclear neutrophils (Giga/L)	–	1.42	1.3	0.6	2.11
C‐reactive Protein (mg/L)	64	90.6	121	160	47
ALAT (UI/L)	–	–	–	51	–
ASAT (UI/L)	–	–	–	106	–
Haptoglobin (g/L)	–	–	–	<0.1	–
LDH (UI/L)	–	–	–	1520	–
Fibrinogen (g/L)	4.45	4.45	4.89	4.89	–
Cephalin clotting time	–	–	–	1.34	–
Kaolin clotting time	–	–	–	1.16	–
Triglyceridemia (mg/dL)	–	–	–	2.85	–
Ferritinemia (*μ*g/L)	–	–	–	1373	498
Potassium (mmol/L)	–	–	–	3.4	–
Proteinuria (g/L)	–	–	–	0.45	–

Within a few hours of antimicrobial therapy, there was an improvement of pulmonary symptoms, yet a deterioration of pancytopenia.

Upon patient's arrival to the internal medicine unit, a bi‐antimicrobial therapy with azithromycin and amoxicillin was started. After 48 h of treatment, new biological deteriorations were observed (Table 1): moderate hepatic cytolysis, cholestasis, hemolysis, low potassium, hypertriglyceridemia, hyperferritinemia, inflammatory syndrome, elevated proteinuria (with normal blood pressure), and deterioration of pancytopenia.

Additional laboratory workup in search for antinuclear antibodies showed very slight amount (one positive antinuclear antibodies reading out of 320). All infectious explorations were negative (blood culture, cytobacteriological examination of urine, parvovirus B 19 serology, and PCR, searching for pneumococcus, legionella, mycoplasma pneumoniae, and chlamydiae, EBV, HCV, TPHA, and VDRL, and HIV serology, and tuberculosis). The patient had immunity against CMV, rubella, and toxoplasmosis.

Deterioration of liver function warranted an abdominal ultrasound which showed isolated and moderate hepatomegaly.

**Figure 2:**
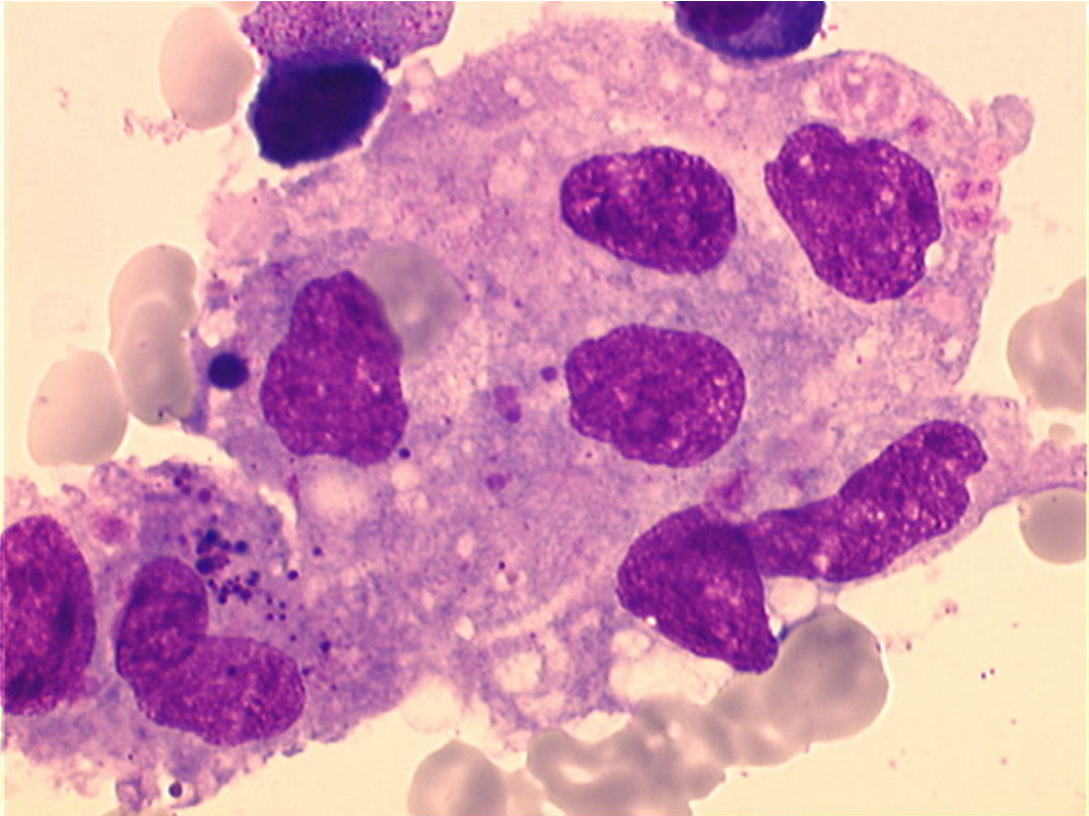
Attached macrophages forming a giant cell with multiple nuclei. Phagocytosis of red blood cells and platelets.

Hemophagocytic syndrome was suspected given the clinicobiological characteristics associating fever, hepatomegaly, pancytopenia, hyperferritinemia, and hypertriglyceridemia. This diagnosis was promptly confirmed by myelogram (Fig. 2).

The myelogram did not show abnormal cells such as Sternberg, or osteoblasts, or osteoclasts. Given the HPS confirmation, parenteral glucocorticosteroids (GC) were started (methylprednisolone at 1 mg/kg). Maturation of fetal lungs was achieved. At the same time, antibiotic spectrum was again enlarged and amoxicillin was replaced with cefotaxime. The clinical and biological evolution became promptly satisfactory. Thromboprophylaxis was started.

In obstetric terms, fetal ultrasound monitoring showed intrauterine growth restriction below the 3rd percentile (i.e., fetal weight: 1548 g at 32 GA + 3 days), with normal fetal and maternal vascular ultrasound. At 38 GA + 4 days, because of the low weight for intrauterine growth restriction (i.e. 2258 g ± 15%), gestational diabetes, and detected oligohydramnios, it was decided to induce labor. The patient gave birth at 38 GA + 6 days, to a girl weighing 2380 g (1st percentile) and in good health.

During the postpartum, oral GC prednisolone was continued for 4 weeks at the dosage of 60 mg per day. A decrease in dosage was scheduled at 3 months postpartum. No HPS relapse occurred after discontinuation of GC and no new autoimmune disease symptoms were found during follow‐up. The newborn's 8‐month clinical examination showed a normal growth without any sign of neurological damage.

## Results

Tables 2 and 3 display the review of HPS peri‐pregnancy data from the literature and our work.

**Table 2:** Displays the results of the comparison between our case and the related literature (first part)

Authors/[biblio]	Gestational age (weeks)	Maternal age (years)	Known risk factors	Prepartum complications	Clinical signs	Laboratory work up	HPS etiology	Study year
Gill et al. 11	18	30	No	Non	Fever, hepatomegaly	Pancytopenia, cytolysis	Unclear	1994
Mihara et al. 10	16	32	No	Non	Fever	Pancytopenia, hyperferritinemia, markedly elevated LDH	EBV	1999
Nakabayashi et al. 13	21	ND	No	Preeclampsia, DIVC, IUGR	Fever	Thrombopenia, leukopenia, cytolysis	Unclear	1999
Chmait et al. 9	29	24	No	DIVC	Adenopathy, fever	Pancytopenia, cytolysis	EBV (postmortem diagnosis)	2000
Yamagushi et al. 17	2nd trimester	ND	No	No	Fever, skin lesions	Pancytopenia, hypertriglycemia, hyperferritinemia, cytolysis	HSV	2005
Pérard et al. 12	22	28	Lupus	Preeclampsia	Fever	Pancytopenia, hypertriglycemia, hyperferritinemia	Lupus	2007
Hahaoka et al. 27	23	33	No	Lymphoma diagnosed	Fever, hepatosplenomegaly	Pancytopenia	B‐cell Lymphoma	2007
Teng et al. 8	23	28	No	Transfusion for anemia compensation and dyspnea improvement	Fever, hepatosplenomegaly	Anemia, thrombopenia, hypertriglycemia	Autoimmune hemolytic anemia	2009
Shukla et al. 28	23	10	No	No	Fever, hepatosplenomegaly	Pancytopenia, hypertriglycemia, hyperferritinemia	Unclear	2011
Arewa et al. 16	21	31	No	No	Fever, jaundice, abdominal pain	Pancytopenia	HIV	2011
Hannebicque Montaigne et al. 5	29	21	Mixed connectivitis (lupus, cryoglobulinemia, Gougerot–Sjogren)	ICU transfer at 22 GA due to vascular failure, bilateral PE, at 25 GA	Fever	Pancytopenia, hyperferritinemia, hypertriglycemia	Lupus	2012
Dunn et al. 14	19	41	Still disease	No	Fever, skin lesions	Cytolysis, anemia, leukopenia, hypertriglycemia, hyperferritinemia	Still Disease	2012
Mayama et al. 19	21	28	No	No	Fever	Pancytopenia hyperferritinemia	Parvovirus B 19	2014
Tumian et al. 15	38	35	No	No	Jaundice	Anemia, thrombopenia, hypertriglycemia, cytolysis	CMV (postmortem diagnosis)	2015
Samra et al. 18	16	36	No	No	Fever, hepatosplenomegaly	Pancytopenia, hyperferritinemia	Unclear	2015
Current	30	44	Raynaud syndrome	Autoimmune	Fever, hepatomegaly	Pancytopenia, hyperferritinemia et hypertriglycemia, cytolysis	History of autoimmune disease	2015

ND, Not documented; IUGR, intrauterine growth retardation; DIVC, disseminated intravascular coagulation; PE, pulmonary embolism; GA, gestational age; CMV, cytomegalovirus; HSV, herpes simplex virus; HIV, human immunodeficiency virus; ICU, intensive care unit; EBV, Epstein–Barr virus.

**Table 3:** Displays the results of the comparison between our case and the related literature (second part)

Authors/[biblio]	Prepartum treatments	Mortality risk factors	C‐section yes/no	Neonatal gestational age (weeks)	Neonatal outcome	Maternal outcome	Study year
Gill et al. 11	Ig IV	Anemia + thrombopenia	No	Full‐term	Alive	Alive	1994
Mihara et al. 10	Glucocorticoides, Ig IV, aciclovir, gabexate	DIVC, age >30	No	35	Alive	Alive	1999
Nakabayashi et al. 13	IgIV	Preeclampsia, DIVC	Yes	29	Alive (respiratory distress)	Alive	1999
Chmait et al. 9	Ig IV, Aciclovir	DIVC	Yes	30	Alive	Dead multi‐organ failure	2000
Yamagushi et al. 17	Glucocorticoides, cyclosporine, aciclovir	Hyperferritinemia	Yes (breech presentation)	37	Alive	Alive	2005
Pérard et al. 12	Glucocorticoides, IgIV	Anemia + thrombopenia + hyperferritinemia	No	30	Alive	Alive (postpartum cerebral hemorrhage)	2007
Hahaoka et al. 27	Chemotherapy R‐CHOP, Cell transplantation	Age >30, anemia + thrombopenia	Yes	28 (fetal distress)	Alive	Alive	2007
Teng et al. 8	Glucocorticoides (treatment failure, improvement after birth)	Anemia + thrombopenia	Yes	29	Dead (respiratory distress)	Alive	2009
Shukla et al. 28	Glucocorticoides, abortion	Anemia + thrombopenia, hyperferritinemia	No	10	Spontaneous miscarriage	Alive	2011
Arewa et al. 16	Antimalaria, HAART	Age >30, anemia + thrombopenia	No	Full‐term	Alive	Alive	2011
Hannebicque Montaigne et al. 5	Ig IV, glucocorticoides	Anemia + thrombopenia + hyperferritinemia	No	38	Alive (neuro postnatal follow‐up, MRI visible cerebral anoxia (asphyxial stigmata)	Alive	2012
Dunn et al. 14	Glucocorticoides	Age >30	Yes (IUGR + twin pregnancy)	30	Alive	Alive	2012
Mayama et al. 19	Glucocorticoides	Hyperferritinemia	No	38	Alive	Alive	2014
Tumian et al. 15	Postpartum onset: glucocorticoides IgIV, cyclosporine	Age >30, DIVC, retard diagnostic	Yes (fetal distress)	38	Alive	Dead multi‐organ failure	2015
Samra et al. 18	Glucocorticoides	Age >30, hyperferritinemia	No	Full‐term	Alive	Alive	2015
Current	Antibiotherapies glucocorticoides	Age >30, hyperferritinemia	No	38	Alive	Alive	2015

IUGR, intrauterine growth retardation; DIVC, disseminated intravascular coagulation; Ig IV, immunoglobulin intravenous; HAART, highly active antiretroviral therapy.

### HPS diagnosis: clinical and biological symptoms

Hemophagocytic syndrome diagnosis was carried out taking into account several specific clinical signs, such as fever >38.5°C, splenomegaly, hepatomegaly, lymphadenopathy, pulmonary infiltrates, erythema, purpura, and neurological evidence. Biological abnormalities were pancytopenia, cholestasis and cytolysis, hyperferritinemia, hypertriglyceridemia, hypofibrinogenemia, and increased LDH 4, 7, 20, 21. According to the literature, fever is the most prevalent clinical sign conveying patient to seek medical help, that is, the fever is often associated with pancytopenia and cytolysis 10 (Tables 2 and 3).

### HPS diagnostic tools

Gold standard diagnostic tool is myelogram which allows identification of hemophagocytosis. Our patient's myelogram showed rich, infiltrated, and benign histiocyte–macrophages. In the absence of histological evidence, a repeated myelogram should be performed 22.

Of note, hemophagocytosis is common in cases of polytransfusion or hematologic diseases and is not regarded as a pathognomonic criterion of HPS 23.

### HPS etiology

Etiological evaluation led to ruling out anyneoplasia 6, 7 (solid malignant tumors, hematologic malignancy). Upon patient questioning, no deterioration of general state was reported. Clinical examination did not reveal any mass or poly lymphadenopathy syndrome. In addition, the myelogram and blood workup did not reveal neoplastic malignant cells. Given the patient's medical history, HPS secondary to autoimmune disease seemed highly likely after excluding any infectious disease. Immune deficiency‐related HPS is very common 4, 6, 15, 16, 17, 18, 19 (45%). The most prevalent pathogens responsible for immune deficiency‐related HPS were reported to be herpesviridae, in particular, CMV, EBV, and HSV. Other less prevalent pathogens were mycobacteria and parasites. Advanced stages of HPS are reported to be secondary to HIV infection 16, 24. In immune deficiency‐related HPS, there is a challenge distinguishing the symptoms induced by pathogens from those secondary to immunosuppression. Indeed, most cases of secondary HPS have been reported in chronic immunosuppression, that is, patients with renal failure, HIV, hematologic or autoimmune disease. Immune deficiency‐related HPS was ruled out given the negative infectious workup.

### HPS treatments

According to the literature, autoimmune related HPS can be treated by the following therapies: GC, intravenous immunoglobulins, methotrexate, and biotherapies 11, 25, 26. The use of GC in our patient allowed reduction in disease progression and complications. Disease progression and its related complications were reduced after GC therapy.

### HPS prognosis

Hemophagocytic syndrome prognosis was positive with satisfactory outcomes for the mother and the fetus. In case of autoimmune disease, the literature has reported only a few cases of positive pregnancy outcomes 10, 13, 16. Fatal pregnancy outcomes have been reported across several reports 8, 9, 15.

## Discussion

### HPS diagnosis

Hemophagocytic syndrome diagnosis is challenging given its rare but serious characteristics often requiring intensive care. The median age for HPS occurrence is 48 yo [35–62 yo] 20 with male predominance 7, 20. There have only been a few reported cases of HPS during pregnancy with sometimes fatal fetal and maternal outcomes 5, 8, 9, 10, 11, 12, 13, 14, 15, 16, 17, 18, 19. According to the literature, the mean age for HPS onset during pregnancy is 31 yo and diagnosis is carried out at the second trimester (around 22 GA) 8, 12, 13, 16, 17, 19, 27, 28.

### HPS etiology

According to the literature, autoimmune disease‐related HPS prevalence is around 7.2% 6, 12. Systemic diseases such as lupus (a prevalence of 2.4%, Wong et al. 29) or Still's disease are the most prevalent cases of secondary HPS 6, 7, 29, 30. In prepartum, our patient presented with moderate peripheral autoimmune thrombocytopenia accompanied by antiribonucleoprotein antibodies and Raynaud's syndrome. In the absence of other events, no specific treatment was started. Nevertheless, the association of the above symptoms with the hormonal changes induced by pregnancy raised the question of vascular‐placental risk. Thus, acetyl salicylate DL‐lysine was prescribed during the entire pregnancy.

Fardet et al. 31 developed a diagnostic score for the adult HPS. It consists of several items: autoimmune disease, maximum temperature, hepatomegaly, splenomegaly, levels of hemoglobin, platelets and leukocytes levels, hyperferritinemia, hypertriglyceridemia, levels of fibrinogen and transaminase, hemophagocytosis found on the bone marrow. This score is a diagnostic aid for gynecologist–obstetrician facing an uncommon but serious pathology often underdiagnosed. It is accessible on http://saintantoine.aphp.fr/score/. According to this score, there was a 96.7% probability that our patient had HPS.

### HPS and pregnancy‐induced risk factors

Pregnancy is a time when the immune system is strongly stimulated and the placenta plays the role of immunological barrier between the fetus and the mother 28. However, this mechanism fails in pregnancy pathologies such as preeclampsia. Given the variable immunological disturbances during pregnancy, it becomes a favorable context to trigger HPS in the presence of additional risk factors such as systemic disease or infection 20. To date, there is no literature on oocyte donation and recipient mother's immune conflict. Nevertheless, we did set forth such likely correlation, that is, the recipient's immunological reaction seemed to be triggered against the presence of unknown genetic matter. Several studies have highlighted an increased rate of pregnancy‐induced hypertension and preeclampsia in patients who underwent in vitro fertilization by oocyte donation versus oocytes of the recipient (OR = 3.3; 95% CI [1.2–8.9]) 32, 33, 34. We believe that this higher pregnancy‐induced hypertension and preeclampsia can be explained by the recipient's immunological reaction triggered against the presence of unknown genetic matter, that is, allogeneic graft. Triggered immunological mechanism of the mother impairs placental implantation and increases maternal systemic resistance leading to preeclampsia and further complications of autoimmune reaction 35. During pregnancy, in the presence of numerous biological signs similar to those of HPS, clinicians should first preclude a differential diagnosis of preeclampsia. Preeclampsia was excluded in our patient case.

### HPS prognosis

Mortality rate linked to the primary or secondary HPS is very high. In HPS, prognosis is poor in 49% of cases and patients with HIV or malignant hemopathy are at higher risk of mortality 6, 7. The literature has put forward the mean premature GA of 30 weeks 7, 11, requiring most likely a C‐section for fetal and maternal salvage in cases of preeclampsia or cerebral hemorrhage 9, 12, 13, 14, 15, 17. Kaito et al. identified the following risk factors of mortality: maternal age >30 years, intravascular disseminated coagulation, anemia associated with thrombocytopenia, cholestasis, elevated ferritin, and β2 microglobulinemia 36.

### HPS treatment

To date, there is no consensus on the best management of either primary or secondary HPS. The overall aim of treatment is to resolve all hydroelectric disorders, transfuse in case of cytopenia, and manage organ failures 37. In addition, it is necessary to treat the cause of HPS: antimicrobial treatment, chemotherapy, or immunomodulators. GC have played a major role in treatment of HPS between 1994 and 2004 and this regardless of the underlying etiology 18.

Even though immunoglobulins are being regarded as the first‐line treatments for HPS, GC have been used as the first‐line treatments in most reported cases 8, 11, 16, 19. Immunoglobulins and cyclosporine have been mostly used for HPS treatment in GC‐resistant cases 18.

## Conclusion

Hemophagocytic syndrome is not well known during pregnancy, yet can be fatal. Mother's and fetus's prognoses are poor and require vital emergency care. HPS diagnosis is a challenge due to variable clinical presentation and nonspecificity of the clinical and biological findings. Mortality, prognosis, and disease progression may be influenced by delay in diagnosis, treatment onset, and HPS etiology. This case and its comparison to the literature showed the absence of consensual diagnosis and management of HPS. Making the right diagnosis in a timely manner during pregnancy seems to be the most significant barrier to treatment and would offer the best prognosis for the patient. Multidisciplinary team work is mandatory to reach prompt diagnosis for such uncommon yet fatal disorder during pregnancy. Clinicians should be alerted when there is an association of clinical and biological signs such as fever, pancytopenia, hyperferritinemia, and hypertriglycemia to suspect HPS and proceed with prompt treatments. To reach consensus on diagnostic criteria for HPS, diagnostic scoring tools, for example, Fardet et al. 31 scoring, as well as novel therapies such as immune modulators combined with biotherapies should be taken into account in further observational studies.

## Authorship

AR, PM, and ZA: contributed to the study design and methodology. AR, PM, ZA, AD, and ELM: contributed to the data interpretation and wrote the manuscript. AR, ELM, AD, CT, and SR: provided patient care and follow‐up, collected patient data, laboratory workup, and interpreted the data. AR and PM: performed the review of literature.

## Conflict of Interest

None declared.
